# Engineering pH responsive fibronectin domains for biomedical applications

**DOI:** 10.1186/s13036-015-0004-1

**Published:** 2015-05-15

**Authors:** Pete Heinzelman, John Krais, Eliza Ruben, Robert Pantazes

**Affiliations:** Department of Chemical, Biological & Materials Engineering, University of Oklahoma, Sarkeys Energy Center, 100 East Boyd Street, Room T-301, 73019 Norman, OK USA; Department of Chemistry & Biochemistry, University of Oklahoma, 73019 Norman, OK USA; Department of Chemical Engineering, University of California-Santa Barbara, 93106 Santa Barbara, CA USA

**Keywords:** Cancer therapy, Epidermal growth factor receptor, Fibronectin, Histidine mutation, Yeast surface display, Biopharmaceutical engineering

## Abstract

**Background:**

Engineered antibodies with pH responsive cell surface target antigen-binding affinities that decrease at the acidic pH (5.5-5.8) within the endosomes have been found to have reduced susceptibility to degradation within the lysosomes and increased serum half-life. Such pH responsive recombinant antibodies have been developed for the treatment of cancer and cardiovascular disease. Engineered tenth type III human fibronectin (Fn3) domains are emerging as a class of target antigen-binding biopharmaceuticals that could complement or be superior to recombinant antibodies in a number of biomedical contexts. As such, there is strong motivation for demonstrating the feasibility of engineering Fn3s with pH responsive antigen binding behavior that could lead to improved Fn3 pharmacokinetics.

**Results:**

A yeast surface-displayed Fn3 histidine (His) mutant library screening approach yielded epidermal growth factor receptor (EGFR)-binding Fn3 domains with EGFR binding affinities that markedly decrease at endosomal pH; the first reported case of engineering Fn3s with pH responsive antigen binding. Yeast surface-displayed His mutant Fn3s, which contain either one or two His mutations, have equilibrium binding dissociation constants (K_D_s) that increase up to four-fold relative to wild type when pH is decreased from 7.4 to 5.5. Assays in which Fn3-displaying yeast were incubated with soluble EGFR after ligand-free incubation in respective neutral and acidic buffers showed that His mutant Fn3 pH responsiveness is due to reversible changes in Fn3 conformation and/or EGFR binding interface properties rather than irreversible unfolding.

**Conclusions:**

We have established a generalizable method for efficiently constructing and screening Fn3 His mutant libraries that could enable both our laboratory and others to develop pH responsive Fn3s for use in a wide range of biomedical applications.

**Electronic supplementary material:**

The online version of this article (doi:10.1186/s13036-015-0004-1) contains supplementary material, which is available to authorized users.

## Background

The tenth type III human fibronectin (Fn3) domain is a useful and versatile scaffold for engineering high affinity binders to a range of protein ligands associated with cardiovascular disease [1] and numerous forms of cancer [2]. The emerging impact of Fn3s in treating these and other health conditions [3, 4] motivates the pursuit of strategies for developing Fn3s with increased serum half-life (t_1/2_). Engineering Immunoglobulin Gs (IgGs) with pH responsive ligand binding affinity has been found to increase t_1/2_ in animal studies by reducing IgG trafficking to and degradation within lysosomes after IgG binding to cell surface target protein ligands and subsequent endocytosis [5, 6]. Despite the positive effect that pH responsiveness can have on IgG pharmacokinetics there are no reports of engineering pH responsive Fn3s for the purpose of increasing t_1/2_. Motivated by the desirable outcomes with pH responsive IgGs, we have established a general approach for engineering pH responsive Fn3s that could have extended t_1/2_s.

Ligand binding scaffolds with pH responsive ligand binding affinity have been engineered using both site-directed [7, 8] and random [7] mutagenesis. Site-directed mutagenesis efforts have consisted of substituting wild type residues, usually residing within regions of the binding scaffold known or expected to be in contact with the ligand, with histidine. The introduction of His mutations is motivated by this amino acid’s sidechain imidazole group having a pKa of approximately 6.0 [9]. In cases where the ionizable His sidechain nitrogen atom is involved in scaffold-ligand interactions, decreasing the pH to below 6.0 can result in nitrogen atom protonation that interferes with scaffold-ligand interaction and reduces binding affinity. It is this potential for pH-mediated reduction in binding affinity that motivated the hypothesis that engineered ligand binding scaffolds that contain site-directed His mutations could have increased t_1/2_s [5, 9].

**Fig. 1:**
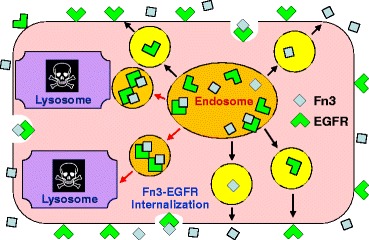
Schematic of cell surface endocytosis and recycling for EGFR and Fn3. Red arrows indicate trafficking of Fn3-EGFR complexes in endosomes (orange circles) to lysosomes for degradation. Black arrows denote movement of transport vesicles (yellow circles) carrying dissociated Fn3 and EGFR molecules to the cell exterior. White indentations denote sites of Fn3-EGFR complex internalization, i.e., sites of endosome formation

With respect to understanding how pH responsiveness arising from His mutations can enhance binding scaffold t_1/2_, one begins by noting that the pH at the cell surface is near neutral (~7.4). Conversely, the pH within the endosomes is acidic (~5.5-5.8). As such, endocytosis of binding scaffold-cell surface receptor complexes results in the complex undergoing a substantial shift in terms of the pH of the surrounding environment. For cases in which binding scaffold His mutations impart pH responsive binding affinity, this pH shift promotes dissociation of scaffold-receptor complexes within the endosome. Whereas large fractions of intact scaffold-receptor complexes are typically trafficked to the lysosome for degradation, both the scaffold and receptor components of the complex are more likely to be recycled to the cell surface, most probably in transport vesicles [9], if the complex dissociates within the endosome (Fig. 1). It is this increased cell surface recycling that is believed to underlie the above noted observed increased *in vivo* t_1/2_ values for pH responsive IgGs [5, 6]. A schematic illustrating both the interplay among the phenomena that govern Fn3 t_1/2_ and the mechanism by which pH responsive ligand binding could increase t_1/2_ appears in Additional file 1: Figure S1.

Yeast surface display is proven as a versatile platform for engineering Fn3s with high affinity and specificity toward a range of protein ligands [2]. Furthermore, both site-directed and random mutagenesis have been successfully employed in using yeast surface display to engineer pH responsive binding scaffolds [7, 10]. These precedents motivated our choosing yeast surface display as our protein engineering platform for the development of pH responsive Fn3s.

There are many examples of applying site-directed amino acid substitution, insertion, or deletion within the Fn3 domain’s three ligand-binding loops to achieve dramatic changes in Fn3 ligand binding specificity and/or binding affinity [2, 11]. These examples motivate seeking to achieve pH responsive ligand binding by targeting His substitutions to these Fn3 loop regions. Fluorescence activated cell sorting (FACS)-based screening of yeast surface-displayed protein libraries has been used to isolate pH responsive Sso7d ligand binding scaffold proteins from a random mutant library [7]. FACS has also been used to enrich pH responsive light (V_L_) and heavy (V_H_) chain antibody variable region domains from yeast-displayed libraries in which His mutations were targeted to the variable domain complementarity determining regions (CDRs) [10]. Additionally, a camelid heavy chain antibody domain (V_HH_) His mutant library in which His mutations were targeted to CDR residues was screened using phage display to yield pH responsive V_HH_ clones containing multiple His substitutions [8]. Combined with the relative simplicity of library construction afforded by the continuous nature of codons representing the residues within a given Fn3 loop, these outcomes suggest that building and screening combinatorial Fn3 binding loop His mutant libraries is a viable strategy for engineering Fn3s with pH responsive ligand binding affinity.

In addition to loop residue substitutions, deletions, and insertions, mutations to Fn3 framework residues have been found to give rise to desired changes in Fn3 ligand binding affinity and specificity [2, 11]. The relatively modest number of framework residues (~70) in a Fn3 domain make one-at-a-time construction and screening of site-directed Fn3 single His mutants a tractable proposition. Regardless of this feasibility, it is desirable to reduce the labor and resources required to identify His substitutions that impart pH responsiveness. Such a reduction could be realized by constructing and screening site-directed Fn3 single His mutants that are predicted to be most likely to possess the desired pH responsive ligand binding affinity. As such, we have employed a structure-guided algorithm [12] to estimate the probability that mutating a given framework residue to His will bring about the desired pH responsive ligand binding affinity and have used these probability estimates to identify a nonrandom, information-guided order for Fn3 single His mutant construction and screening.

The design of the structure-guided algorithm, which quantifies the number of neighboring amino acids for each Fn3 residue [12], that we have used to identify a nonrandom order for Fn3 framework residue site-directed Fn3 single His mutant construction and screening, has been motivated by two hypotheses. The first hypothesis is that pH responsive ligand binding affinity arising from Fn3 framework residue His mutations will be the result of ionization-induced changes in the tertiary structure of the Fn3 domain rather than the creation of unfavorable Fn3-ligand interactions that are created by His protonation. The second hypothesis is that mutating amino acid sidechains that are buried within the Fn3 domain’s hydrophobic core will be much more likely to disrupt Fn3 tertiary structure in a way that abolishes ligand binding than to impart pH responsiveness. Taken together, these hypotheses led us to develop a structure-guided algorithm that, as discussed below, outputs the number of neighboring residues for each amino acid represented in the Fn3 crystal structure.

In applying the number of neighbors outputs of this algorithm to determine a nonrandom order for site-directed single His mutant construction and screening, we assume that substituting His for residues with the greatest number of neighbors has a high probability of leading to disruptions in Fn3 tertiary structure that abolish ligand binding. We further posit that substituting His for residues having the smallest number of neighbors has a low probability of giving rise to pH responsive ligand binding. This low probability is presumed by virtue of these low number of neighbor residues participating in a relatively small number of interactions with other amino acids within the Fn3 domain. This low number of interactions is expected to reduce the likelihood that protonation of a substituted His sidechain will perturb Fn3 tertiary structure. The above assumptions motivated our choosing to prioritize Fn3 framework residues with an intermediate number of neighbors in determining a nonrandom order for site-directed single His mutant construction and screening.

Our choice of a parent Fn3 for pH responsiveness engineering was motivated both by a desire to bind a cell surface receptor that has clinical relevance and our wishing to have a parent receptor-binding Fn3 with high receptor binding affinity at neutral pH. The latter criterion arises from the observation that His mutations that give rise to pH responsiveness tend to reduce ligand binding affinity under both acidic and neutral conditions [8–10]. High ligand binding affinity for the parent Fn3 helps protect against the possibility that affinity reductions at neutral pH will be large enough to reduce binding affinity to cell surface ligands to a level that is not therapeutically relevant. An epidermal growth factor receptor (EGFR)-binding Fn3, previously engineered by yeast surface display, known as Clone A (CA), satisfied both of the above criteria; EGFR has been targeted in treating colon, neck, and breast cancer [13] and CA binds EGFR with single-digit nanomolar affinity [11].

## Results

### Structure-guided quantification of Clone A (CA) framework amino acid residue neighbors

The precedent [2, 11] for modifying Fn3 ligand binding loops to modulate ligand binding specificity and affinity made constructing site-directed loop residue single His mutants and/or combinatorial loop residue His mutant libraries a logical starting point for seeking to demonstrate that His mutations can give rise to Fn3 variants with pH responsive ligand binding affinity. Given however, that the ability of framework mutations to influence Fn3 binding affinity and specificity is understudied relative to the ability of loop mutations to modulate Fn3 ligand binding properties, we were intrigued by the potential to assess the degree to which framework His mutations might enable pH responsive binding. As such, we chose to initiate our pH responsiveness engineering pursuits by constructing and screening a collection of site-directed CA framework residue single His mutants.

As discussed in the Background section, we developed and applied a structure-guided algorithm, which is based on an established method for quantifying amino acid neighbors [12], to identify CA residues that possess an intermediate number of neighbor residues. The algorithm, which defines neighboring residues as having sidechain β carbons that are within 10 Ǻ of each other, was applied to the structure for the wild type tenth type III human fibronectin domain (PDB entry 1FNA).

The respective ligand binding loop sequences for the wild type and EGFR-binding CA Fn3 domains are highly diverged. Conversely, the framework residue identities for these two Fn3s differ at only one position (Additional file 1: Figure S2). Given our emphasis on quantifying neighbor amino acids for framework residues and the generally surface exposed nature of loop residues, which is expected to result in loop residues making a relatively small contribution to framework residue neighbor amino acid counts, we felt justified in using the wild type Fn3 crystal structure in quantifying neighbors for CA framework residues.

The CA framework residue analysis found the average number of framework residue neighbors to be 14.5 with a standard deviation of 4.5 (Additional file 1: Table S1). We defined residues with respective low, intermediate, and high numbers of neighbors as being below, within, or above one standard deviation of the mean number of neighbors. Using these criteria, the respective numbers of framework residues with low, intermediate, and high numbers of neighbors were 10, 41, and 16 (Additional file 1: Table S1).

Calculated over all of the 91 Fn3 residues represented in 1FNA the average number of neighbors was 14.0 with a standard deviation of 4.4 (Additional file 1: Table S1). Of the 24 loop residues, 7 have low numbers of neighbors while 15 and 2 have respective intermediate and high numbers of neighbors. This shift toward lower values in the number of neighbors distribution for loop residues relative to framework residues is in accord with the above stated expectation that the generally surface exposed nature of loop residues will result in their having fewer neighbors than residues comprising the Fn3 framework. The above noted high level of conservation of framework residue primary sequence holds across the majority of engineered Fn3 domains. As such, it is reasonable to assume that the framework residue classifications determined here can be applied in determining an information-guided, nonrandom order in which framework residue site-directed single His mutants could be constructed and screened for almost any Fn3 of interest.

### Construction and screening of site-directed CA single His mutants

We arbitrarily chose 20 CA residues from the intermediate number of neighbors group for site-directed mutagenesis to His. Additionally, in order to test the hypothesis that mutating residues with large numbers of neighbors would result in abolition of CA binding to EGFR we chose 15 CA residues from the high number of neighbors group for site-directed His mutagenesis.

**Fig. 2:**
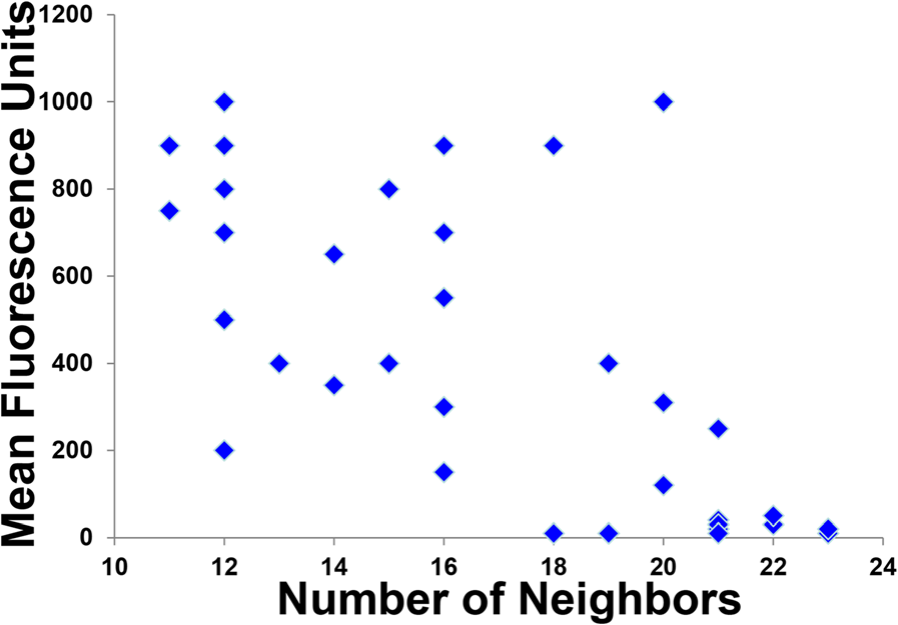
Flow cytometry mean fluorescence units (MFU) versus number of neighbors for 35 CA single His variants. MFU values obtained for yeast incubated with 1 nM EGFR at pH 7.4

All 35 of the single His mutants were displayed on the yeast surface at high levels as measured by flow cytometry. The observed inverse relationship between number of neighbors and EGFR binding affinity at pH 7.4 as measured by flow cytometry is illustrated by the scatterplot of Fig. 2. As shown by the flow cytometry MFU (mean fluorescence unit) data in Additional file 1: Table S2, the inverse relationship between number of neighbors and EGFR binding affinity was relatively pH-independent as respective MFU values at pH 7.4 and pH 5.5 varied by 20 % or less for 28 of the 35 single His mutants.

Eleven of the fifteen high neighbors group His mutants had MFU values that were similar to those for the negative control at both pH 7.4 and pH 5.5 (Additional file 1: Table S2). Three of the other four mutants in the high neighbors group had notably reduced MFU values relative to wild type CA at both pHs. Only one of the 15 His variants in this collection possessed binding affinity comparable to wild type CA. In contrast to the high number of neighbors group, 12 of the 20 intermediate neighbors group His mutants possessed MFU values that were at least 50 % of the value measured for wild type CA at either pH 7.4 or both pH 7.4 and pH 5.5.

**Fig. 3:**
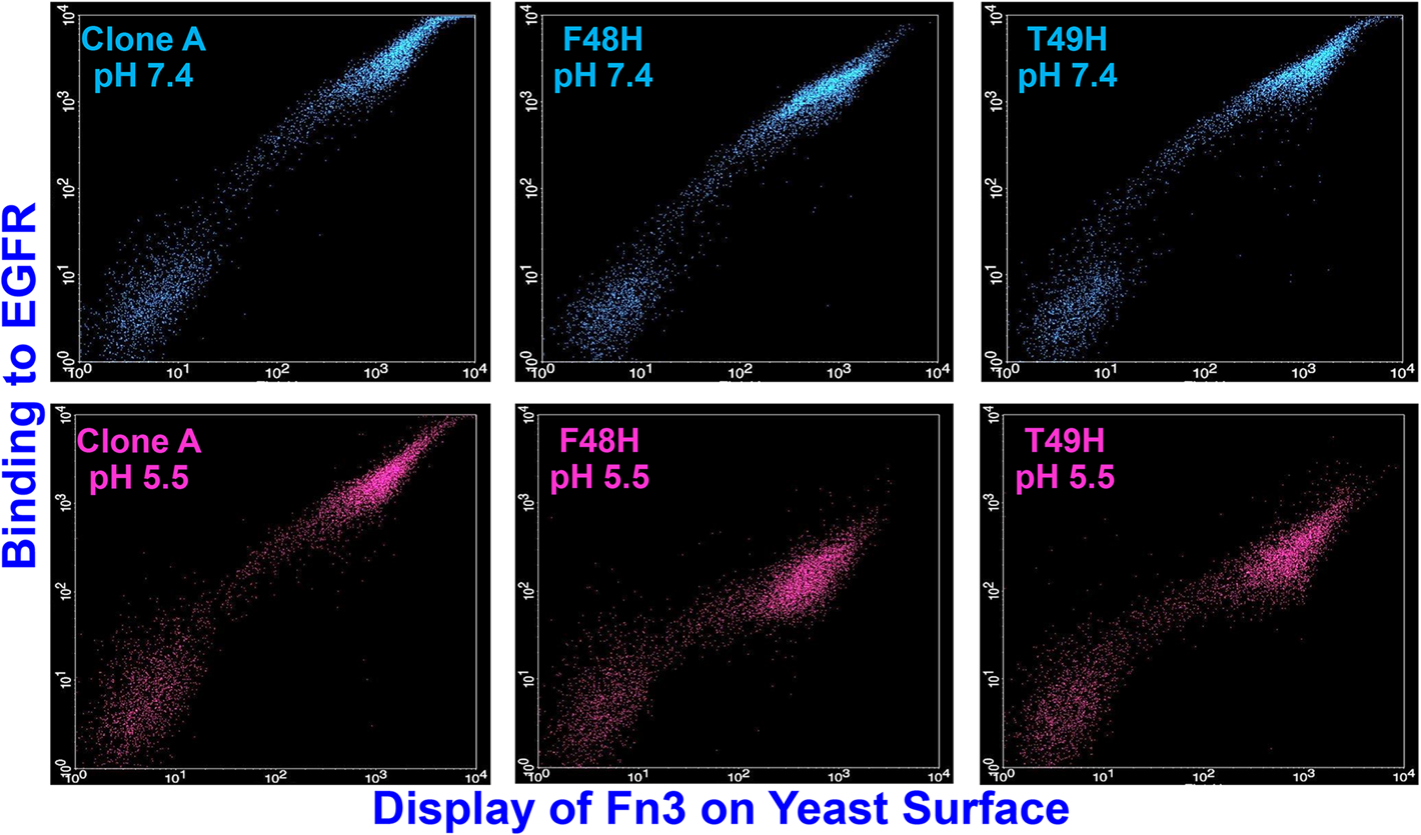
Flow cytometry dot plots for yeast-displayed wild type, F48H, and T49H Fn3 CA. Cells incubated with 10 nM biotinylated EGFR. Y-axis denotes phycoerythrin fluorescence corresponding to EGFR binding. X-axis denotes FITC fluorescence corresponding to *myc* tag yeast surface display

Two of the single His mutants, F48H and T49H, possessed pH responsiveness screen MFU values that were reduced by 75% or more at pH 5.5 relative to pH 7.4 (Fig. 3 & Additional file 1: Table S2). The appreciable MFU reductions at pH 5.5 for these two mutants distinguished them from the other His mutants in the library; the His mutants with the third and fourth greatest relative reductions in MFU, Y36H and A57H, possessed relative MFU decreases of approximately 40% (Additional file 1: Table S2). The appreciable separation of the F48H and T49H mutant relative MFU reduction values from those for the other His mutant library members, along with the MFU values for the F48H and T49H mutants being either equivalent or not unduly reduced relative to wild type CA at pH 7.4, motivated our focusing on the mutations at the F48 and T49 positions in further Fn3 His mutant binding affinity characterizations.

Given the precedent for multiple His mutations imparting highly pH responsive binding affinity [5, 8, 10], we constructed a F48H/T49H double His mutant to be analyzed along with the F48H and T49H single His mutants in binding affinity titrations. We note here that an unintended E47D mutation was present in both the F48H and T49H single mutants due to an error in DNA oligo design during the cloning process. This E47D mutation was intentionally carried over into the F48H/T49H double His mutant but was not incorporated into the parent CA construct used in the flow cytometry affinity titrations described below.

### Yeast-displayed wild type & His mutant Clone A binding affinity titrations

**Fig. 4:**
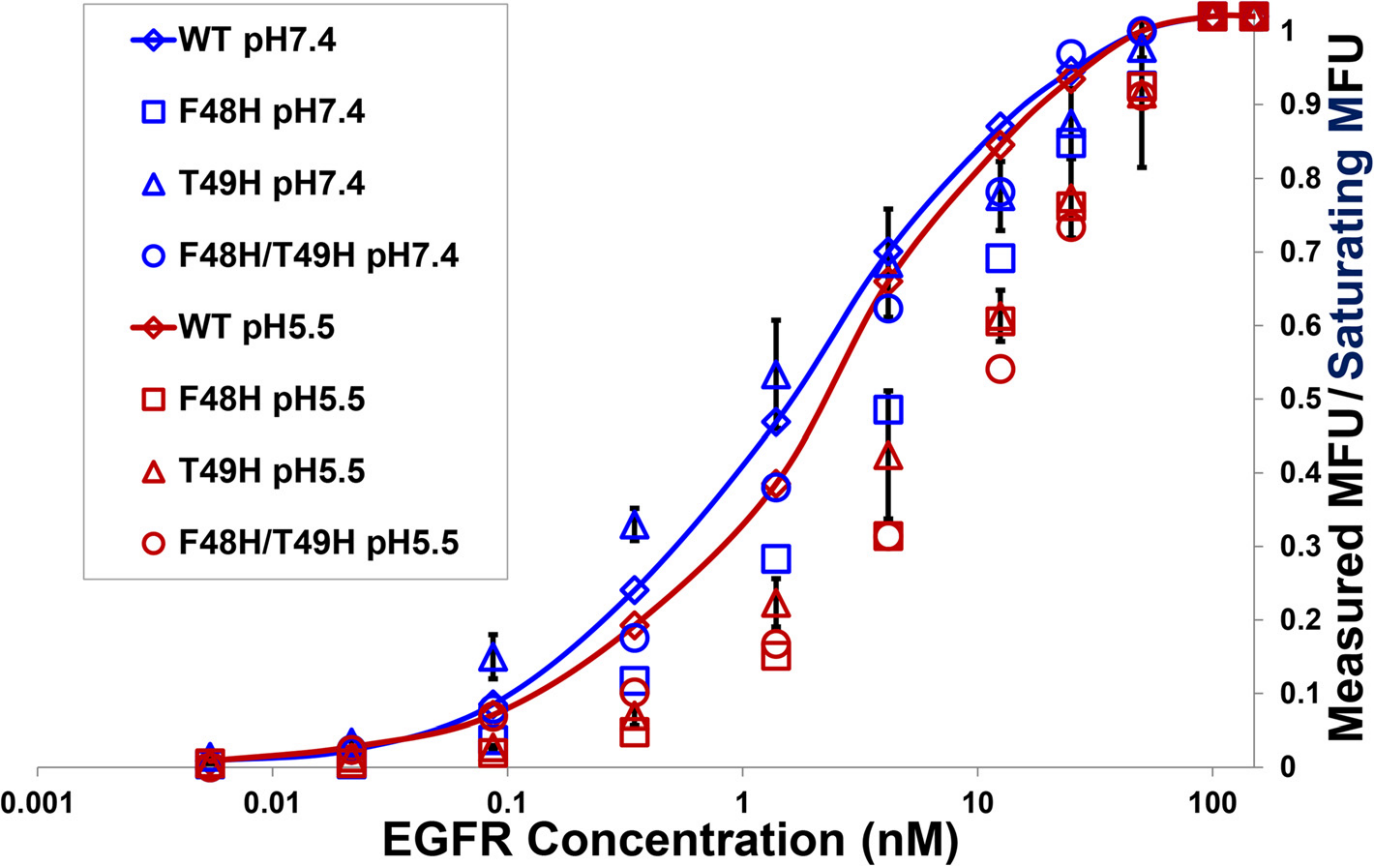
Binding affinity titration curves for yeast-displayed wild type and His variant CA proteins. Y-axis denotes ratio of Mean Fluorescence Unit (MFU) values measured via flow cytometry at a given EGFR concentration to MFUs measured at saturating EGFR concentration (50–100 nM) for respective wild type and His variant CAs. X-axis denotes EGFR concentration. Measured:saturating MFU ratios for EGFR binding at pH 7.4 denoted by blue markers; MFU ratios for EGFR binding at pH 5.5 denoted in red. Data points represent average values for two independent trials. Respective blue and red lines connect data points for wild type CA binding to EGFR at pH 7.4 and pH 5.5. Error bars, which appear for T49H mutant data, denote standard deviation for two independent trials

Our CA single His mutant screening dataset consisted of single flow cytometric binding measurements at pH 7.4 and pH 5.5 for each mutant. As such, prior to initiating soluble expression and purification of Fn3 domains for use in surface plasmon resonance (SPR) binding affinity measurement, we wished to further verify that the F48H, T49H, and F48H/T49H CA mutants possessed pH responsive EGFR binding affinity. We also wanted to confirm that CA His mutant pH responsiveness was not accompanied by unduly large decreases in EGFR binding affinity at neutral pH. We achieved these objectives by carrying out flow cytometric binding affinity titrations in which the binding of yeast surface-displayed wild type CA and the three His mutants to EGFR was measured across a range of EGFR concentrations (Fig. 4).

The binding affinity titration results verified the increased pH responsiveness of all three CA His mutants relative to wild type CA (Table 1). T49H had the most desirable EGFR binding property profile of the three His mutants. This mutant possessed EGFR binding affinity (K_D_ = 1.3 +/− 0.5 nM) that was similar to wild type (K_D_ = 1.7 +/− 0.1 nM) at pH 7.4 and a ratio of K_D_s (pH 5.5:pH 7.4) that was increased more than four-fold, i.e., from 1.4 +/− 0.2 to 6.3 +/− 0.7. The respective F48H and F48H/T49H mutants, both of which possessed mildly decreased EGFR binding affinity relative to wild type at pH 7.4, had approximate two- and four-fold K_D_ ratio increases relative to wild type CA.

**Table 1:** EGFR binding affinity parameters for yeast-displayed wild type CA and His variants

Fn3 Domain	K_D_ pH 5.5 (M)	K_D_ pH 7.4 (M)	K_D_ Ratio (5.5/7.4)
Wild Type Clone A	(2.4 +/− 0.4) •10^−9^	(1.7 +/− 0.1) •10^−9^	1.4 +/− 0.2
F48H	(1.1 +/− 0.4) •10^−8^	(4.8 +/− 0.8) •10^−9^	2.3 +/− 0.4
T49H	(8 +/− 2) •10^−9^	(1.3 +/− 0.5) •10^−9^	6.3 +/− 0.7
F48H/T49H	(1.5 +/− 0.4) •10^−8^	(3 +/− 1) •10^−9^	5.2 +/− 0.4

Error bars denote standard deviations for two independent trials in which K_D_s were determined by fitting data obtained in respective binding affinity titrations conducted at pH 5.5 and pH 7.4 and carried out simultaneously. For each clone, two independent K_D_ ratio values were obtained by dividing the K_D_ values that were calculated based on the data for each of the two independent pairs of EGFR binding affinity titrations

**Fig. 5:**
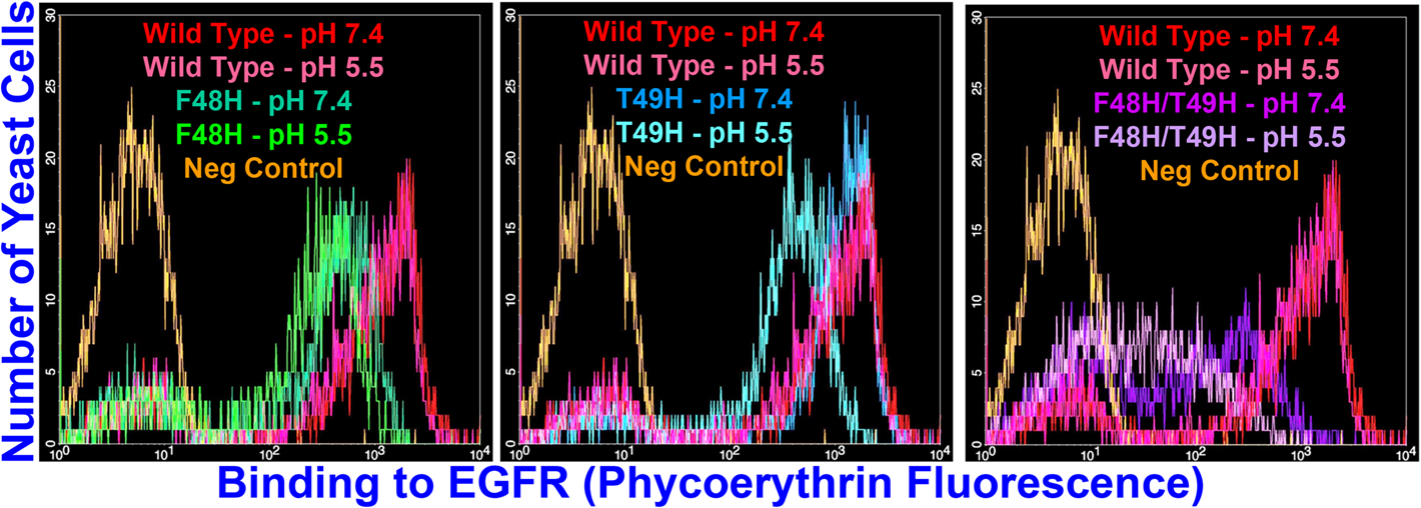
Binding histogram overlays showing pH responsiveness for respective F48H, T49H, and F48H/T49H CA mutants. X-axis denotes phycoerythrin fluorescence corresponding to EGFR binding. Y-axis denotes number of yeast cells in sample analyzed with given level of EGFR binding. Wild type (red-pH 7.4, pink-pH 5.5) incubated with 1.5 nM EGFR in all figure panels. F48H (dark green-pH 7.4, bright green-pH 5.5) incubated with 4 nM EGFR, T49H (blue-pH 7.4, cyan-pH 5.5) incubated with 1.5 nM EGFR, and F48H/T49H (purple-pH 7.4, lavendar-pH 5.5) incubated with 12 nM EGFR. Unlabeled, negative control yeast population histograms appear in orange in all figure panels. As shown in Additional file 1: Figure S3, F48H and F48H/T49H were displayed at lower levels than T49H and wild type CA

The EGFR binding histogram overlays of Fig. 5, which present EGFR binding data collected at the EGFR concentrations that most strongly broke out differences in binding affinity at respective neutral and acidic pH for the CA His mutants, further demonstrate the appreciable decreases in mutant binding affinity at pH 5.5 relative to pH 7.4 that are reflected by the K_D_ data of Table 1. The pH 5.5 His mutant histograms are notably shifted toward lower fluorescence values relative to the pH 7.4 His mutant histograms in all of these overlays. Conversely, the pH 5.5 and pH 7.4 histograms for wild type CA are nearly identical.

As shown by the surface display level measurement flow cytometry histograms of Additional file 1: Figure S3, the F48H mutation mildly decreased CA yeast surface display level while display for the F48H/T49H double mutant was notably reduced. The surface display level for the T49H mutant was similar to that for wild type CA.

### Verifying reversibility of pH dependent decrease in binding affinity for yeast-displayed wild Clone A His mutants

The need for clinically relevant pH responsive Fn3s to be capable of undergoing multiple cycles of ligand binding, endocytosis, and recycling to the cell exterior motivated experiments to address the question of whether irreversible structural changes, such as partial or complete unfolding of the protein, contributed to the observed reductions in EGFR binding affinity for the yeast surface-displayed CA His mutants at pH 5.5. We addressed this question by incubating wild type and His mutant CA-displaying yeast in either pH 7.4 or pH 5.5 buffer (phosphate buffered saline with 1 mg/mL bovine serum albumin, PBS-BSA) for one hour in the absence of ligand. Subsequently, both the neutral and low pH buffer incubation cell samples were washed and incubated with various concentrations of biotinylated EGFR in pH 7.4 PBS-BSA.

**Fig. 6:**
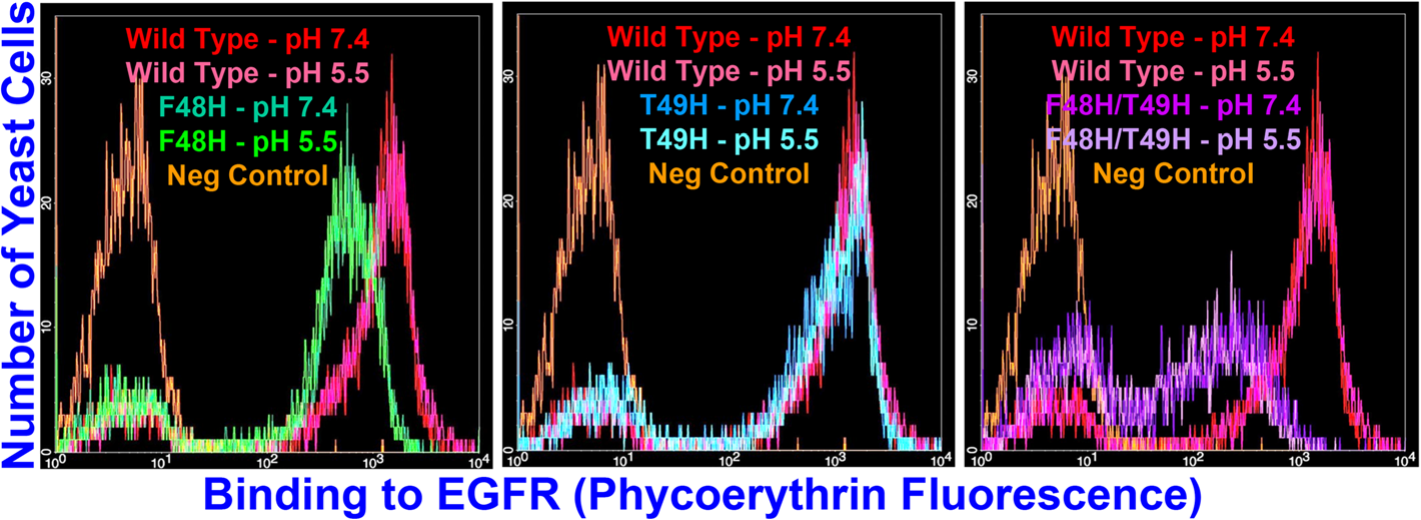
Binding histogram overlays showing that CA His mutants do not undergo irreversible conformational changes at low pH. X-axis denotes phycoerythrin fluorescence corresponding to EGFR binding. Y-axis denotes number of yeast cells in sample analyzed with given level of EGFR binding. Wild type (red-pH 7.4, pink-pH 5.5) incubated with 1.5 nM EGFR in all figure panels. F48H (dark green-pH 7.4, bright green-pH 5.5) incubated with 4 nM EGFR, T49H incubated with 1.5 nM EGFR (blue-pH 7.4, cyan-pH 5.5), and F48H/T49H (purple-pH 7.4, lavendar-pH 5.5) incubated with 12 nM EGFR. All incubations with EGFR performed in pH 7.4 buffer. The preceding pH values in parentheses and those appearing in the figure denote ligand-free incubation buffer pH values. Unlabeled, negative control yeast population histograms appear in orange in all figure panels. As shown in Additional file 1: Figure S3, F48H and F48H/T49H were displayed at lower levels than T49H and wild type CA

As shown in Table 2 and Fig. 6, wild type and His mutant CA binding affinity toward biotinylated EGFR was independent of the pH at which the CA-displaying yeast were incubated prior to ligand exposure. These results show that the pH responsiveness of the yeast-displayed CA His mutants is due to pH dependent, reversible changes in protein conformation and/or ligand binding interface properties rather than the induction of irreversible changes in protein structure under acidic conditions.

**Table 2:** Flow cytometry MFU values for CA-displaying yeast incubated with biotinylated EGFR after ligand-free buffer incubation

Fn3 Domain	EGFR Concentration (nM)	MFU - pH 5.5 Pre-incubation	MFU - pH 7.4 Pre-incubation
Wild Type Clone A	1.5	1370 +/− 100	1340 +/− 100
Wild Type Clone A	4.0	1850 +/− 160	1650 +/− 100
F48H	4.0	650 +/− 20	620 +/− 80
F48H	12	1270 +/− 40	1200 +/− 100
T49H	1.5	1200 +/− 40	1250 +/− 120
T49H	4.0	1460 +/− 120	1600 +/− 30
F48H/T49H	12	150 +/− 10	160 +/− 20
F48H/T49H	25	220 +/− 30	240 +/− 20

Wild type and His variant CA-diplaying yeast were incubated with various concentrations of biotinylated EGFR in pH 7.4 PBS-BSA after one hour incubation in ligand-free PBS-BSA with pH adjusted to either 5.5 or 7.4. EGFR concentrations were chosen to match or be similar to those at which reductions in CA EGFR binding affinity at pH 5.5 relative to pH 7.4 were most clearly broken out in K_D_ titrations. Values represent averages for two independent trials. Error bars denote standard deviations

### Expression and purification of wild type and His mutant CA in *E. Coli*

Having verified that the CA His mutants were more pH responsive than wild type CA, not unduly compromised with respect to binding affinity at pH 7.4, and not susceptible to irreversible structural changes under acidic conditions, we proceeded with soluble expression and purification of the wild type and all three His variant CA proteins. As shown in the SDS-PAGE gel analysis of Additional file 1: Figure S4, single step purification of *E. coli* expression culture cell extracts using Co^2+^ affinity resin enabled isolation of reasonably pure (~70-90%) soluble CA wild type and His mutant proteins, all of which contained C-terminal His_6_ tags. Our desire to employ the soluble Fn3s in SPR experiments, which provide higher quality data when carried out with highly purified proteins than when executed using lower purity protein samples, motivated us to further enrich the soluble CA wild type and His variant proteins via size exclusion chromatography (SEC).

SEC analysis at pH 7.4 (Additional file 1: Figure S5) of post-Co^2+^ affinity resin-purified wild type and His mutant CA proteins showed that wild type CA existed as a mixture of various molecular weight (MW) isoforms: monomer (~12.5 kDa), dimer (~25 kDa), and high MW oligomer. All of the His mutants appeared almost exclusively as high MW oligomers.

The high MW oligomers eluted in the SEC column void volume fraction, a phenomenon generally observed for proteins or protein complexes that possess column gel matrix migration properties similar to those of protein species with MWs that are greater than the column’s upper MW limit (~660 kDa). Attempts to better resolve this oligomer’s MW via native PAGE analysis were unsuccessful; the high MW complexes did not migrate through the polyacrylamide gel matrix in a way that enabled MW estimation (data not shown). All three of the wild type CA MW isoforms, as well as the three His mutant CA oligomers, migrated at the same MW (~12.5 kDa) upon reducing SDS-PAGE analysis (Additional file 1: Figure S6). The yields of post-SEC purified protein for the wild type and His mutant CA isoforms are presented in Additional file 1: Table S3.

Accurate comparison of SPR-measured wild type and His mutant CA binding affinities or avidities requires that the proteins being characterized are in identical oligomeric states. As such, prior to initiating SPR experiments we sought to verify the stability of the wild type and His mutant CA high MW oligomers at acidic pH. As shown in Additional file 1: Figure S7, neither wild type nor His mutant CAs appear as high MW oligomers in the chromatograms when SEC is carried out at pH 5.5. Wild type CA elutes as a mixture of dimeric and monomeric isoforms whereas there are no discernible elution peaks for any of the CA His mutants.

We posit that multiple factors contributed to the contrast between wild type and His mutant CA proteins in terms of the respective observed presence and absence of defined elution peaks in the pH 5.5 SEC chromatograms. The SDS-PAGE analysis of Additional file 1: Figure S4 shows that the *E. coli* culture expression levels for the CA mutants were lower than for wild type CA. The SEC column was loaded with equal volumes of post-Co^2+^ affinity resin-purified CA protein solution. As such, the mass of CA His mutant protein injected onto the SEC column was less than the mass of injected wild type CA. Additionally, it is possible that the high MW CA His mutant oligomers dissociate into a mixture of lower MW oligomers, dimers, and monomers and that isoforms of a given MW are heterogeneous with respect to protein structural conformation. The combination of such MW and conformational heterogeneity and relatively low protein loading would be expected to result in considerable diffusion of the CA His mutants across the SEC column and prevent the SEC chromatograms from featuring well-defined elution peaks.

Regardless of what factors were responsible for the absence of peaks in the pH 5.5 CA His mutant chromatograms it is clear that neither the wild type nor the His mutant CA high MW oligomers are stable at acidic pH. Given this observation we chose not to pursue the planned SPR experiments. Although the inability to quantify EGFR binding affinities for soluble wild type and His mutant CA proteins is not a desirable outcome the appreciable yields and high purity achieved for both wild type and His variant CA bodes well for high level production of Fn3s developed in the context of future pH responsive Fn3 engineering pursuits.

## Discussion

Our structure-guided algorithm for determining a nonrandom order of Fn3 framework amino acid single His mutant construction and screening enabled efficient identification of Fn3 CA His mutants with pH responsive binding affinity. The 15 high number of neighbors mutants were constructed and screened for the purpose of assessing the utility of our classification algorithm in predicting Fn3 His mutant retention of ligand binding affinity at neutral pH. As such, we can state that it has been possible to create multiple pH responsive CA variants by way of constructing and screening just 20 site-directed single His mutants.

This efficient isolation of pH responsive CA variants groups our single His mutant construction and screening strategy among random mutant [7] and site-directed His mutant [7, 8, 10] library screening as proven approaches for engineering pH responsive recombinant protein variants. Unlike both random and site-directed mutant library screens, our screening strategy is not subject to inconclusive outcomes in which one cannot determine whether an inability to enrich pH responsive library members arises from such clones not existing or their not being isolated due to inadequate screening method fidelity and/or insufficient library sampling. As such, there are contexts, such as making an initial demonstration of pH responsive binding for a given protein of interest, in which our single His mutant screening approach is a more appropriate protein engineering strategy than seeking to enrich pH responsive clones from large libraries.

Also under the umbrella of desirable aspects of our single His mutant screening approach, there are cases in which one might want to engineer pH responsive variants of Fn3s that bind to different epitopes on the same antigen [11]. In such situations, the time and materials cost savings that can arise from constructing and screening site-directed His mutants in a nonrandom order, as opposed to constructing and screening a set of single His mutants comprised of respective members with substitutions at each of the ~ 100 amino acid positions that constitute a given Fn3, become quite considerable.

An additional opportunity for other researchers to benefit from employing the methods that we have utilized in this work arises from the high degree of framework residue conservation across different engineered Fn3s. This high degree of conservation should make the outcomes of our CA number of neighbors analysis useful for determining a nonrandom order of single His mutant construction and screening for essentially any engineered tenth type III human fibronectin of interest. As such, even researchers who are not particularly well-versed in applying protein structure-based calculations can benefit from our number of neighbors algorithm by way of reducing the time and materials costs associated with engineering pH responsive Fn3s. Also in the vein of our CA His mutant engineering results streamlining future pH responsive Fn3 engineering efforts, the conservation of the F48 and T49 residues across the majority of engineered Fn3 frameworks motivates exploring the possibility that His mutations at these positions could be common enablers of pH responsive ligand binding in the background of engineered Fn3s other than CA.

Prior studies of soluble, purified CA binding to EGFR do not report the presence of multiple MW isoforms [11]. As such, we were surprised to find that both wild type and His mutant CA existed in monomeric, dimeric, and oligomeric states after Co^2+^ affinity resin purification. Both our work and prior CA expression efforts [11] utilized similar *E. coli* expression strains, expression plasmids, and expression conditions. As such, it is likely that wild type CA dimers and/or oligomers were present in purified CA samples prepared by others but were never detected as a result of single-step metal ion affinity chromatography, with no subsequent SEC step, being used for CA purification.

The instability and potential MW and conformational heterogeneity of Fn3 oligomers prevents Fn3s that oligomerize from being viable candidates for clinical translation. As such, Fn3s that are being considered as parent scaffolds for yeast display engineering should be expressed as soluble proteins, metal ion affinity chromatography purified, and analyzed by SEC prior to initiating yeast surface display engineering efforts. In this vein, we have been encouraged to learn that others’ observations of multimerization of Fn3-like proteins [13, 14] have spurred efforts to design recombinant Fn3-like protein libraries comprised of members that have high loop sequence diversity but low propensity for multimerization.

In seeking insights regarding the structural basis of the observed pH responsive binding for the F48H, T49H, and F48H/T49H CA mutants we examined both the 1FNA crystal structure and a homology model of our own construction in which the native Fn3 binding loops were modified to match those of wild type CA. As can sometimes be the case with such structural examinations, we were not able to formulate any particularly strong hypotheses to explain the protein property of interest, in this case pH responsive EGFR binding. We can however, confidently state that the consecutive nature of the pH sensitizing F48H and T49H mutations is coincidental; the respective F48H and T49H sidechains are oriented at 180° relative to each other and have nonoverlapping sets of neighbor amino acids.

Based on our CA homology model, it appears that the sidechain of the residue at position 47, which as noted in the Results section is a Glu residue in wild type CA but is mutated to Asp in all of the pH responsive mutants, would interact with the sidechain of a His residue that has been substituted for the native Thr at CA position 49 (Additional file 1: Figure S8). There is not however, any apparent interaction between a substituted His residue at position 48 and the sidechain, whether it be Asp or Glu, of the residue appearing at position 47. Given this observation and the observed pH responsive binding of the F48H CA mutant, it is unlikely that the E47D substitution is a determinant of CA His mutant pH responsive ligand binding.

The feasibility of engineering Fn3s that can bind to almost any antigen of interest is well-established [2]. As such, the generality of the approach that we have used to create pH responsive CA His mutants raises the exciting possibility of developing a collection of pH responsive Fn3s that bind to a wide range of different respective cell surface target antigens. Provided that an ample number of the members of such a group of pH responsive Fn3s can be expressed and purified in a stable monomeric form, this set of engineered, pH responsive antigen binders could enable a broad based assessment of the utility of leveraging reduced Fn3 trafficking to the lysosomes and the concomitant increase in cell surface recycling as a strategy for improving Fn3 pharmacokinetics that could be used as either a complement or an alternative to Fn3 PEGylation [15] for increasing t_1/2_.

## Conclusions

Combining a structure-guided approach for determining a nonrandom order of Fn3 single His mutant construction and screening with a yeast surface display-based screening protocol has enabled efficient identification of His mutations that give rise to Fn3s with pH responsive ligand binding. To our knowledge, the pH responsive EGFR-binding Fn3 His mutants isolated in this work are the first-ever engineered Fn3s with pH responsive ligand binding affinity. We are optimistic regarding the prospects for our setting this Fn3 engineering precedent to enable both our laboratory and others to develop pH responsive Fn3s that can have an impact in the development of new and effective regimens for treating cancer, cardiovascular disease, and other health conditions.

## Methods

### Structure-guided quantification of number of neighbors for CA framework amino acids

The structure-guided algorithm we developed for quantifying the number of neighbors for CA amino acids was based on an established hydrogen bond potential function [12]. Our algorithm defines neighboring residues as having sidechain β carbons that are within 10 Ǻ of each other. For glycine, which does not have a sidechain β carbon, the α carbon atom being within 10 Ǻ of another amino acid’s β carbon was used to define neighboring residues.

The distances between all pairs of representative atoms were calculated to determine the number of neighbors for each amino acid in the structure for the wild type tenth type III human fibronectin domain (PDB entry 1FNA). Amino acids with neighbor counts within one standard deviation of the average were classified as having an intermediate number of neighbors. Respective amino acids with neighbor counts one standard deviation above and below the average were classified as having high and low numbers of neighbors.

### Construction of Clone A site-directed His mutants

Site-directed His mutations were introduced into the CA Fn3 gene via overlap extension PCR using the wild type CA yeast surface display plasmid as template in conjunction with outer upstream primer *ConSqLt* (*5′-CTACTCTTTGTCAACGACTAC-3′*) and outer downstream primer *ConSqRt (5′-CATGGGAAAACATTTTTTACG-3′)*. These respective oligonucleotides prime approximately 80 bases upstream and downstream of the CA gene. Overlap PCR reactions were digested with NheI and BamHI and ligated into similarly digested pCTCON yeast surface display backbone vector. Ligation products were transformed into chemically competent DH5-alpha *E. coli* cells (New England Biolabs) and His mutant sequences verified using the above primers. Site-directed His mutant surface display plasmids that sequenced correctly were transformed into *S. cerevisiae* surface display strain EBY100 made competent using the Zymo Research Frozen EZ-Yeast Transformation II kit.

### Yeast surface-displayed Clone A His mutant flow cytometric screening

Individual EBY100 colonies carrying CA surface display plasmids were picked into 5 mL of SC-CAA media and grown overnight at 30°C before being induced for 24 h in 5 mL of SG-CAA at 30°C. Induced cultures were resuspended in phosphate buffered saline (PBS) with 1 mg/mL bovine serum albumin (BSA) prior for flow cytometric analysis of EGFR binding.

Recombinant human EGFR ectodomain produced in Chinese hamster ovary cells, a gift from the laboratory of Dr. Greg Adams at the Fox Chase Cancer Center, was biotinylated using biotin-NHS (Pierce) and exchanged into pH 7.4 PBS using a Zeba Spin desalting column (Pierce). Flow cytometric screening for pH responsiveness was performed by incubating yeast cells with 1 nM biotinylated EGFR ectodomain in PBS-BSA at both pH 7.4 and pH 5.5 for 90 min at 25°C. These incubations included anti-myc IgY (Life Technologies) at a concentration of 20 μg/mL. Cells were washed and secondary labeling was performed on ice by incubation with FITC-conjugated goat anti-IgY (Jackson Immunoresearch) at 20 μg/mL and streptavidin-phycoerythrin (Jackson Immunoresearch) at 10 μg/mL in pH 7.4 PBS-BSA. FITC and phycoerythrin fluorescence signals were measured using a Becton Dickinson FACSort flow cytometer.

For yeast surface display wild type and His variant CA affinity titrations CA-displaying yeast were incubated with various concentrations of biotinylated EGFR for 90 min at 25°C. Cells were washed and secondary labeling was performed on ice by incubation with streptavidin-phycoerythrin in pH 7.4 PBS-BSA as above. Mean fluorescence values (MFUs) were determined for the entire population, i.e., both displaying and nondisplaying yeast cells. Equilibrium binding dissociation constant (K_D_) values were determined using the Excel solver function and a 3-parameter fit (K_D_, MFU_range_ and MFU_min_) as described [16].

In assays to verify that His variant CA mutant pH responsiveness was not the result of irreversible changes in protein conformation wild type or His variant CA-displaying yeast were incubated in either pH 5.5 or pH 7.4 PBS-BSA for 1 h at 25°C. Yeast cells were then washed once in pH 7.4 PBS-BSA and incubated with various concentrations (Table 2) of biotinylated EGFR in pH 7.4 PBS-BSA for 90 min at 25°C. Cells were washed and secondary labeling was performed on ice by incubation with streptavidin-phycoerythrin in pH 7.4 PBS-BSA. MFU values for the entire yeast cell population were measured via flow cytometry as described above.

### Wild type & His mutant Clone A expression & purification

Wild type and His mutant CA genes were cloned into the NcoI and BamHI sites of the pET28a expression vector with C-terminal His_6_ tags and expressed in BL21(DE3) *E.coli* cells using LB media containing 25 μg/mL of kanamycin. Five mL overnight cultures were used to inoculate 500 mL cultures. Five-hundred mL cultures were grown at 37°C to A_600_ of 0.3 before induction with 0.5 mM IPTG. CA proteins were expressed for 5 h at 37°C. Cells were pelleted at 4,000 rpm (Beckman Coulter Avanti J-26S centrifuge, JLA 8.1 fixed angle rotor) for 30 min and resuspended in lysis buffer (20 mM Tris–HCl, 150 mM NaCl, pH 8.0) at a ratio of 10 mL of buffer to 1 g of wet cell pellet. Cells were lysed by three passages through a C3-Emulsiflex (Avestin) at a pressure of 15,000 psi. Lysates were clarified by 30 min of centrifugation at 15,000 rpm (Beckman Coulter Avanti J-26S centrifuge, JA 25.50 fixed angle rotor).

CA protein in the lysate supernatants was purified by affinity chromatography Talon Co^2+^ resin (Clontech). Lysate was incubated with 1 mL of Talon resin pre-equilibrated with lysis buffer at 25°C for 30 min with gentle agitation. Resin was next packed into an empty 10 mL column and washed with 30 mL of 20 mM Tris–HCl, 150 mM NaCl, 10 mM Imidazole, pH 8.0 before final elution with 20 mM Tris–HCl, 150 mM NaCl, 500 mM Imidazole, pH 8.0.

For the second protein purification step, i.e., size exclusion chromatography, samples were run over a Superdex 75 10/300 GL size exclusion column (GE Healthcare) using an AKTA Pure FPLC system (GE Healthcare). The column was calibrated using a Gel Filtration LMW Calibration Kit (GE Healthcare). Post-Talon resin purified CA samples were injected in 500 μL volumes and isocratic elution with PBS carried out at a flow rate of 0.5 mL/min. Samples were sterile filtered using a 0.22 μM polyethersulfone syringe filter (Pall Life Sciences) and CA concentrations determined via microscale Bradford assay (BioRad). Low pH SEC was carried out as above using 20 mM Na citrate, 150 mM NaCl, pH 5.5, as the column running buffer.

## Additional file

Additional file 1:
**Is a pdf containing all of the Supplementary Figures and Tables, as well as corresponding descriptive captions, referred to in the main text.**
