# Supplementary material for: Engineering pH responsive fibronectin domains for biomedical applications
Source: J Biol Eng. 2015 May 15;9:6. doi: 10.1186/s13036-015-0004-1 (PMC4477602; doi:10.1186/s13036-015-0004-1)
Supplement: Additional file 1: — Is a pdf containing all of the Supplementary Figures and Tables, as well as corresponding descriptive captions, referred to in the main text. [file 13036_2015_4_MOESM1_ESM.pdf]

Additional File One - Supplementary Material

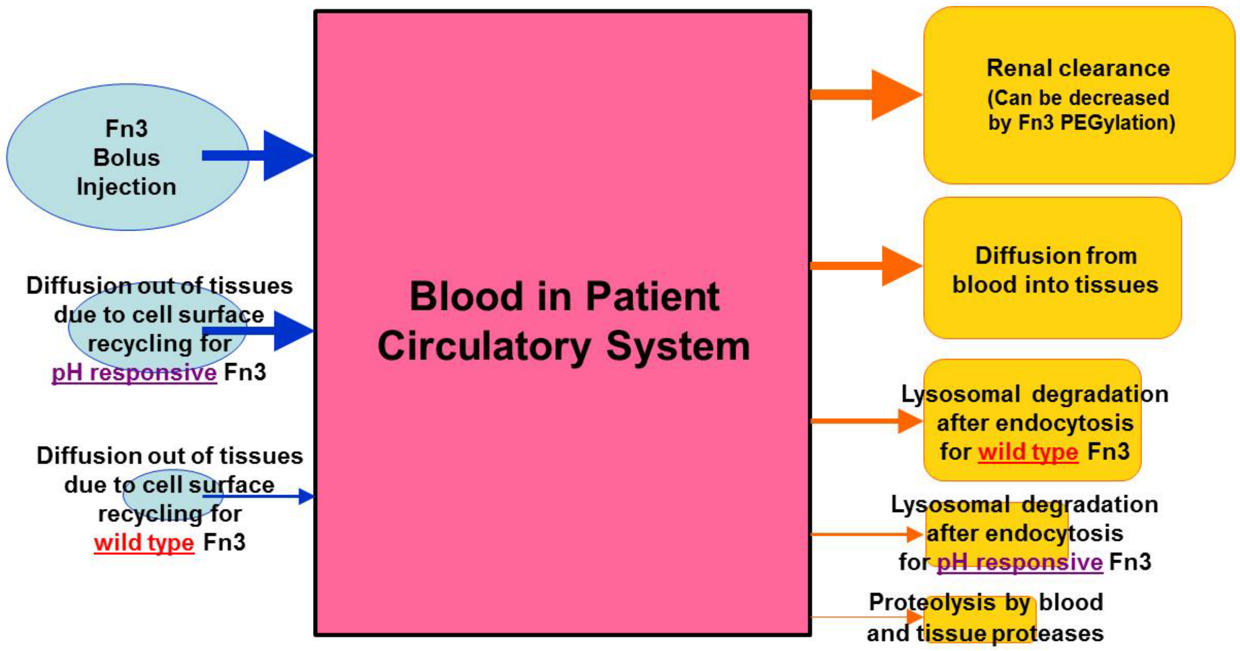

Supplementary Figure 1. Schematic depicting mechanism by which increased cell surface recycling could increase $t_{1/2}$ for pH responsive Fn3s relative to their wild type counterparts. Blue arrows and ovals denote phenomena that increase or help maintain Fn3 concentration in blood. Phenomena that decrease concentration of Fn3 in blood are denoted by orange arrows and rounded rectangles.

>Wild_Type_Fn3
VSDVPRDLEVVAATPTSLLISW**DAPAVTVRY**YRITYGETGGNSPVQEFTVP**GSKST**ATIS
GLKPGVDYTITVYAVT**GRGDSPASSK**PIS**I**NYRTEIDKP

>Fn_3_Clone_A
VSDVPRDLEVVAATPTSLLISW**FDYAVTY**YRITYGETGGNSPVQDFTVP**GWIST**ATIS
GLKPGVDYTITVYAVT**DNSRWPFRST**PIS**T**NYRTEIDKP

Supplementary Figure 2. Amino acid sequences for wild type and Clone A Fn3 domains. Ligand binding loops highlighted in blue. Framework amino acid residue difference (I90T) denoted in green.

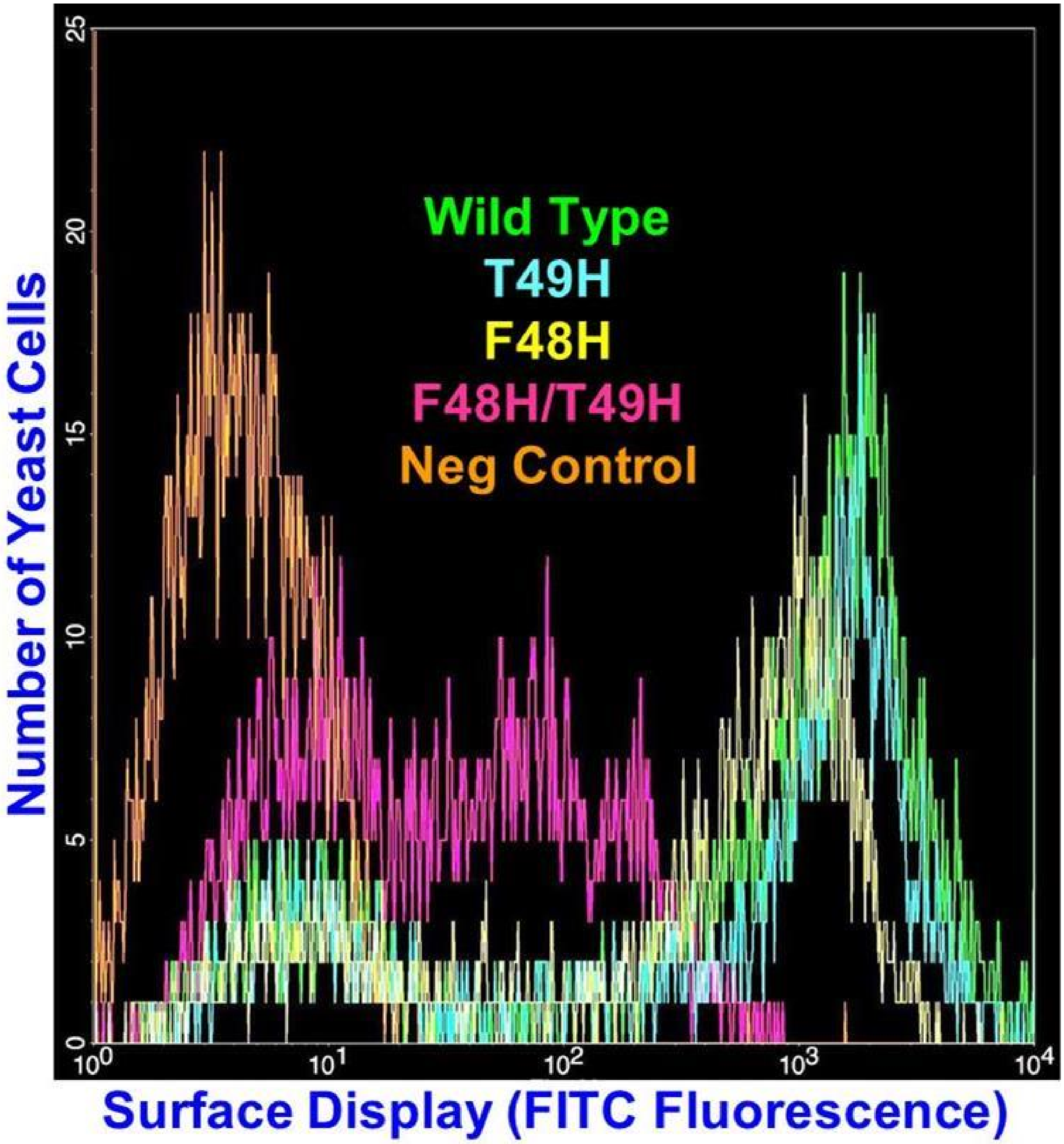

Supplementary Figure 3. Flow cytometry histograms for wild type and CA His variant displaying yeast incubated with primary anti-*myc* antibody and secondary FITC-conjugated goat anti-mouse antibody. Antibody fluorescence (X-axis) correlates with CA yeast surface display level. Y-axis denotes number of cells in the population of analyzed yeast with a given level of wild type or His variant CA display.

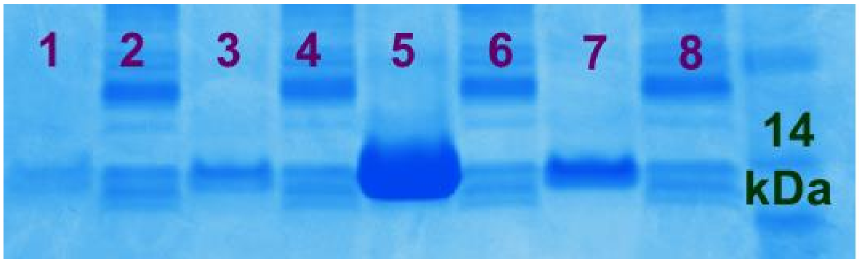

Supplementary Figure 4. SDS-PAGE analysis of Co^{2+}-resin affinity purified Fn3s. All Fn3 bands appear near anticipated monomeric CA molecular weight of 12.5 kDa. Lanes [left-to-right]: 1) F48H/T49H resin eluate 2) F48H/T49H resin flowthrough 3) F48H resin eluate 4) F48H resin flowthrough 5) Wild type CA resin eluate 6) Wild type CA resin flowthrough 7) T49H resin eluate 8) T49H resin flowthrough

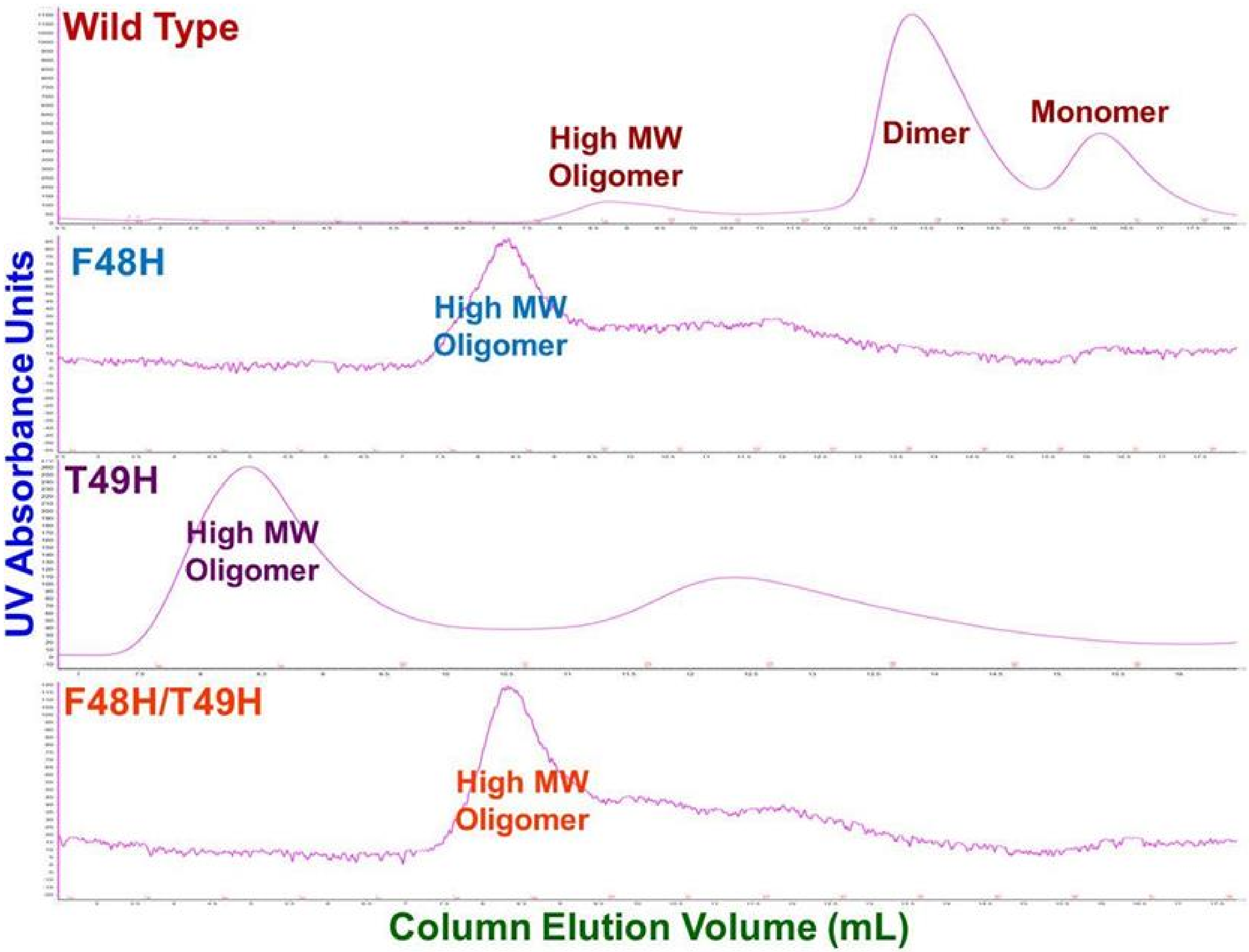

Supplementary Figure 5. SEC chromatograms for wild type and His mutant CA proteins fractionated at pH 7.4. Y-axis (UV absorbance units) and X-axis (column elution volume) scales vary among chromatograms. Respective wild type CA high MW, dimer, and monomer peaks appear at approximately 8 mL, 13 mL, and 16 mL. CA His mutant high MW oligomer peaks appear at 8 mL.

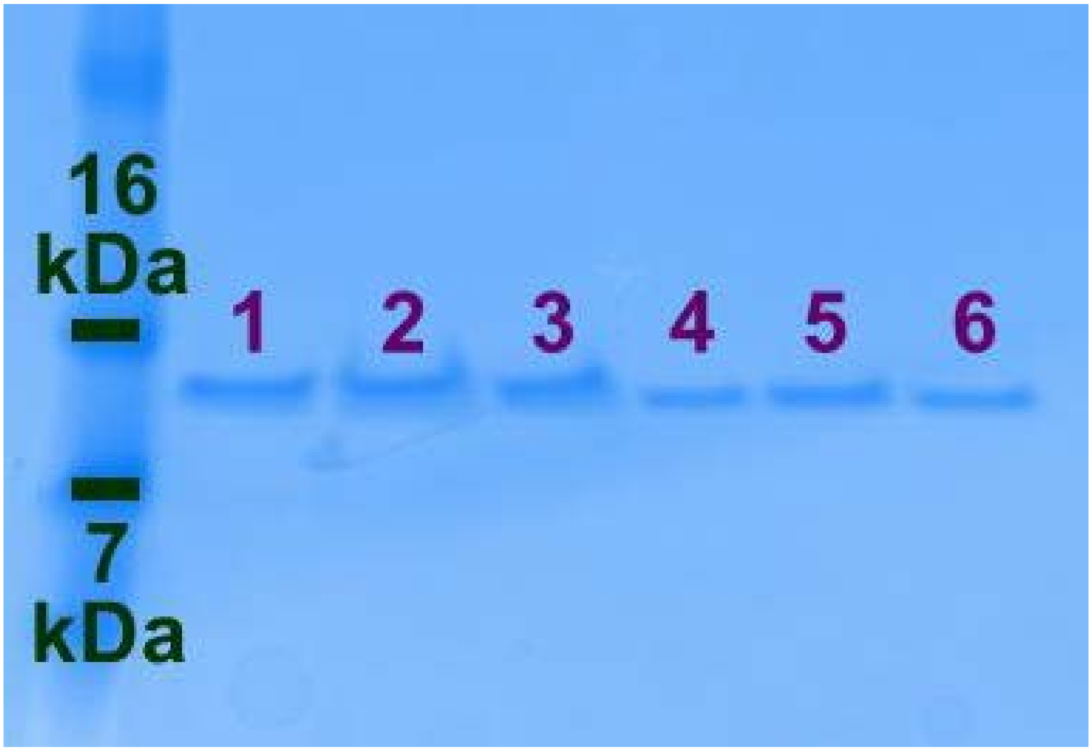

Supplementary Figure 6. Reducing SDS-PAGE analysis for SEC-purified wild type CA monomer, dimer, and oligomer fractions and His mutant oligomer fractions. All isoforms migrate near anticipated molecular weight of 12.5 kDa. Lanes [left-to-right]: 1) Wild type high MW oligomer 2) Wild type dimer 3) Wild type monomer 4) F48H oligomer 5) T49H oligomer 6) F48H/T49H oligomer

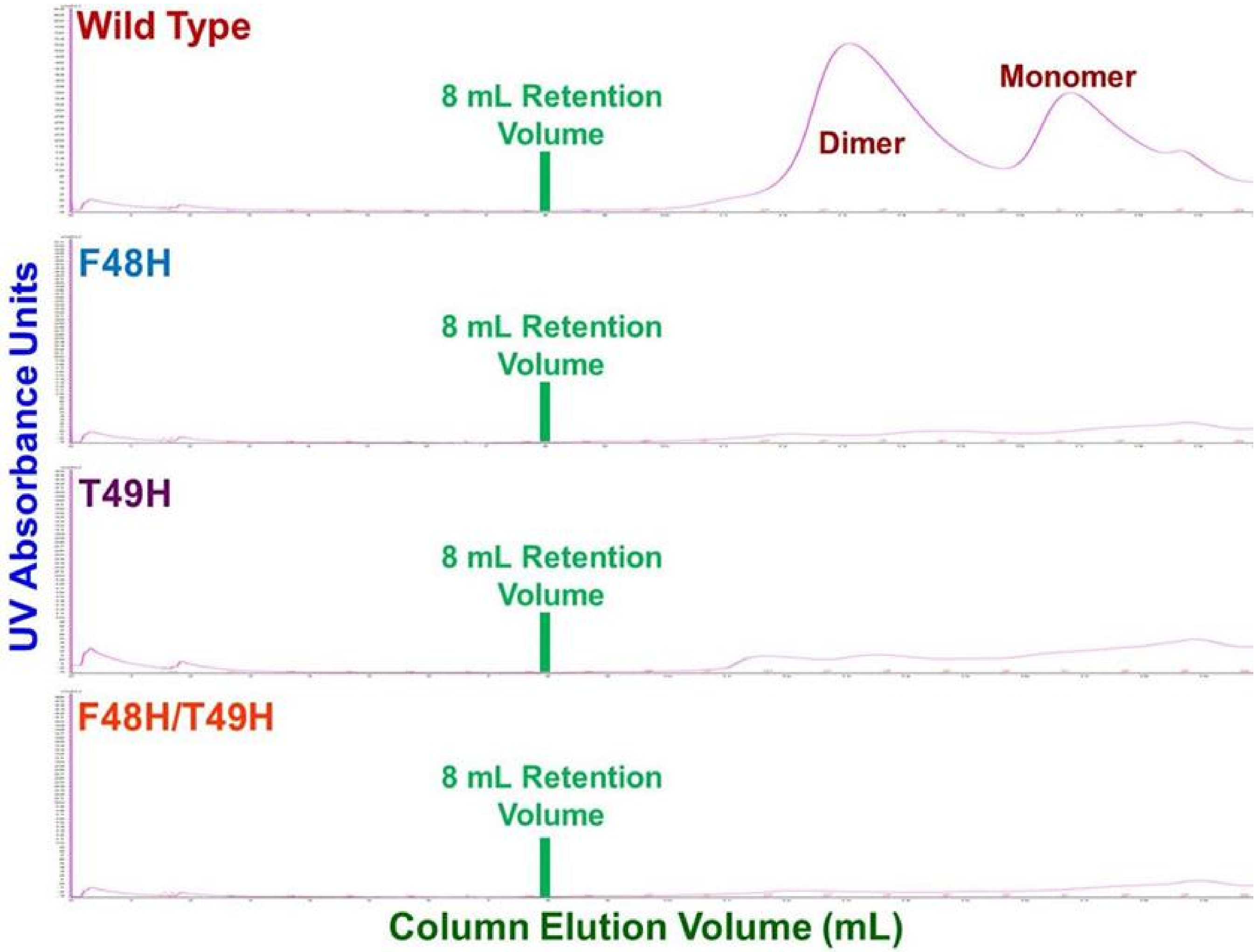

Supplementary Figure 7. SEC chromatograms for wild type and His mutant CA proteins fractionated at pH 5.5. Y-axis (UV absorbance units) scales vary among chromatograms. Respective wild type CA dimer and monomer peaks appear at approximately 13 mL and 16 mL. The 8 mL retention volume value at which high MW oligomers were observed for SEC carried out at pH 7.4 is denoted by vertical green bars in all four chromatograms.

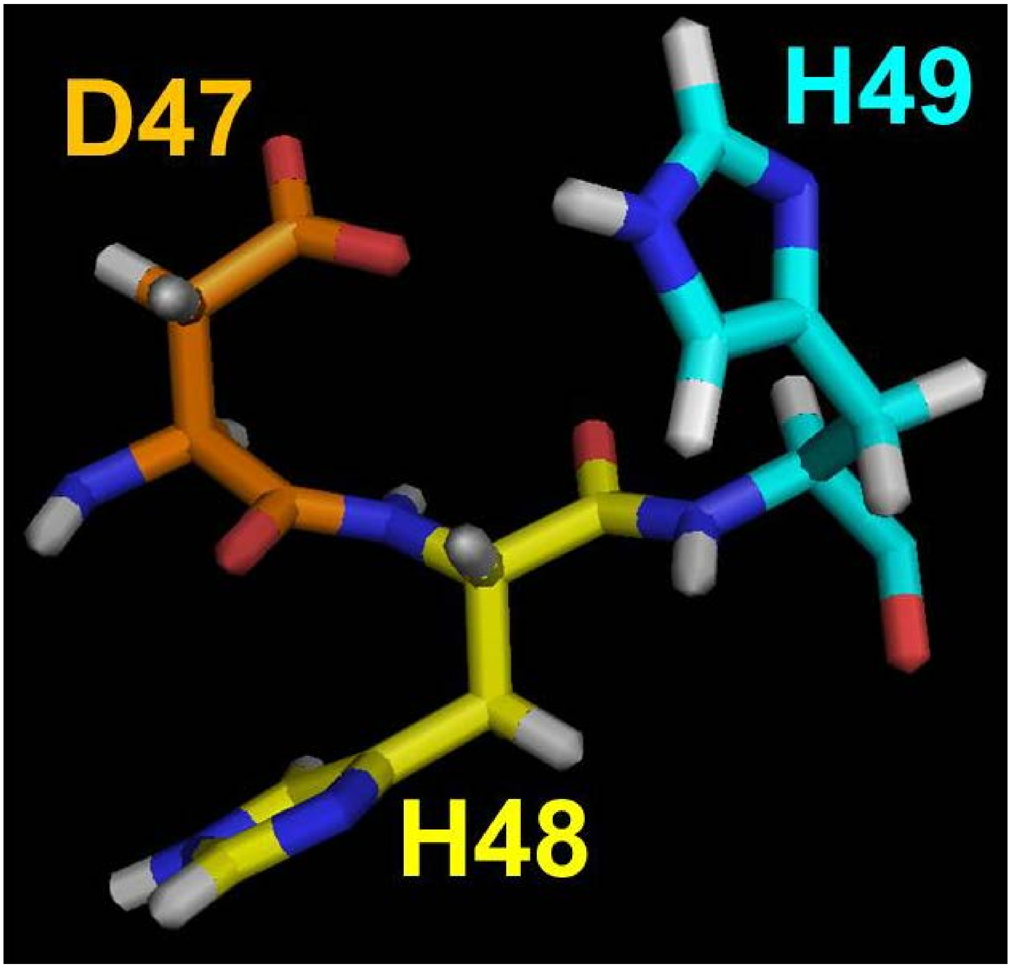

Supplementary Figure 8. Zoom view of residues D47, H48 and H49 in homology model for the CA F48H/T49H mutant based on PDB entry 1FNA. Carbon atoms for respective D47, H48 and H49 residues appear in orange, yellow and cyan. Nitrogen atoms appear in blue, oxygen atoms in red and hydrogen atoms in white. Figure illustrates that D47 and H49 sidechains are proximal to each other but distant from the H48 sidechain. Figure created with Pymol.

Supplementary Table 1. Numbers of neighboring amino acids for all residues represented in PDB entry 1FNA. Ligand binding loop residues denoted in bold italics.

Residue	Number of Neighbors	Classification
Ile70	23	High
Val72	23	High
Ile20	22	High
Ala74	22	High
Leu18	21	High
Trp22	21	High
Tyr32	21	High
Tyr36	21	High
Ile59	21	High
Val29	20	High
Tyr68	20	High
Tyr92	20	High
Thr94	20	High
Gly37	19	High
Val50	19	High
Leu62	19	High
Tyr73	19	High
Ser84	19	High
Ile34	18	Intermediate
Ala57	18	Intermediate
Ser85	18	Intermediate
Leu8	17	Intermediate
Pro25	17	Intermediate
Ser55	17	Intermediate
Val75	17	Intermediate
Val10	16	Intermediate
Ala13	16	Intermediate
Tyr31	16	Intermediate
Thr35	16	Intermediate
Val66	16	Intermediate
Thr69	16	Intermediate
Thr71	16	Intermediate
Thr76	16	Intermediate
Ile88	16	Intermediate

Ile90	16	Intermediate
Thr16	15	Intermediate
Leu19	15	Intermediate
Ala24	15	Intermediate
Arg30	15	Intermediate
Arg33	15	Intermediate
Glu38	15	Intermediate
Gly52	15	Intermediate
Ser21	14	Intermediate
Gln46	14	Intermediate
Asp67	14	Intermediate
Pro15	13	Intermediate
Ser17	13	Intermediate
Thr39	13	Intermediate
Lys54	13	Intermediate
Pro87	13	Intermediate
Arg93	13	Intermediate
Thr14	12	Intermediate
Asp23	12	Intermediate
Val45	12	Intermediate
Glu47	12	Intermediate
Phe48	12	Intermediate
Thr49	12	Intermediate
Lys63	12	Intermediate
Gly65	12	Intermediate
Ser89	12	Intermediate
Arg6	11	Intermediate
Glu9	11	Intermediate
Ala12	11	Intermediate
Val27	11	Intermediate
Thr28	11	Intermediate
Pro51	11	Intermediate
Thr56	11	Intermediate
Thr58	11	Intermediate
Pro64	11	Intermediate
Gly77	11	Intermediate
Asp80	11	Intermediate
Glu95	11	Intermediate
Gly41	10	Intermediate

Arg78	10	Intermediate
Val11	9	Low
Ser60	9	Low
Gly79	9	Low
Lys86	9	Low
Asn91	9	Low
Asp7	8	Low
Ala26	8	Low
Gly40	8	Low
Ser43	8	Low
Ser53	8	Low
Gly61	8	Low
Pro82	8	Low
Ala83	8	Low
Ile96	8	Low
Pro44	7	Low
Ser81	6	Low
Asn42	5	Low

Supplementary Table 2. Flow cytometry mean fluorescence unit (MFU) values for CA single His mutant pH responsive EGFR binding screen.

Residue	Number of Neighbors	Classification	EGFR Binding at pH 5.5 (MFU)	EGFR Binding at pH 7.4 (MFU)
Wild Type	N/A	N/A	800	900
Neg Cont	N/A	N/A	10	10
Ile70	23	High	10	10
Val72	23	High	10	20
Ile20	22	High	30	30
Ala74	22	High	30	50
Leu18	21	High	40	40
Trp22	21	High	20	20
Tyr32	21	High	30	30
Tyr36	21	High	150	250
Ile59	21	High	10	10
Tyr68	20	High	70	120
Tyr92	20	High	800	1000
Thr94	20	High	220	310
Val50	19	High	10	10
Leu62	19	High	10	10
Tyr73	19	High	450	400
Ile34	18	Intermediate	10	10
Ala57	18	Intermediate	550	900
Val10	16	Intermediate	150	150
Ala13	16	Intermediate	700	700
Thr69	16	Intermediate	850	900
Thr71	16	Intermediate	450	550
Ile90	16	Intermediate	350	300
Thr16	15	Intermediate	750	800
Glu38	15	Intermediate	300	400
Gln46	14	Intermediate	600	650
Asp67	14	Intermediate	300	350
Arg93	13	Intermediate	400	400
Thr14	12	Intermediate	700	700
Val45	12	Intermediate	400	500
Glu47	12	Intermediate	1000	1000
Phe48	12	Intermediate	50	200
Thr49	12	Intermediate	200	900

Lys63	12	Intermediate	800	800
Ala12	11	Intermediate	700	750
Glu95	11	Intermediate	700	900

Supplementary Table 3. Post-SEC purified yields (mg pure Fn3/liter culture) for wild type and His mutant CA proteins fractionated at pH 7.4. ND indicates that an enrichable elution peak for the given isoform was not present in any of the eluted SEC fractions (Supplementary Figure 5).

**Fn3 Domain**	**Monomer (mg/L)**	**Dimer (mg/L)**	**High MW Oligomer (mg/L)**
Wild Type Clone A	70	20	20
F48H	ND	ND	20
T49H	ND	ND	40
F48H/T49H	ND	ND	20
